# The effectiveness and safety of acupuncture for chemotherapy-induced peripheral neuropathy: A systematic review and meta-analysis

**DOI:** 10.3389/fneur.2022.963358

**Published:** 2022-10-03

**Authors:** Zhonghang Xu, Xingbo Wang, Yuanyu Wu, Chengbing Wang, Xuedong Fang

**Affiliations:** China-Japan Union Hospital of Jilin University, Jilin University, Changchun, China

**Keywords:** chemotherapy-induced peripheral neuropathy, acupuncture, systematic review, meta-analysis, physiotherapy

## Abstract

**Objectives:**

This systematic review and meta-analysis aimed to evaluate the effectiveness and safety of acupuncture on chemotherapy-induced peripheral neuropathy (CIPN).

**Methods:**

We searched for relevant randomized controlled trials (RCTs) in PubMed, Cochrane Library, and Embase databases from their inception to 1 April 2022. The Functional Assessment of Cancer Therapy/Gynecologic Oncology Group-Neurotoxicity (FACT/GOG-Ntx), Brief Pain Inventory-Short Form (BPI-SF), the European Organisation for Research and Treatment of Cancer Quality of Life Questionnaire-Core30 (EORTC QLQ-C30), Numerical Rating Scale (NRS), and adverse events were the outcome measures. All studies had at least one of these outcome measures. Mean differences (MDs) with 95% confidence intervals (CIs) were assessed in the meta-analysis using the RevMan 5.3 software.

**Results:**

Five studies were included in the analysis. The results showed that acupuncture and placebo acupuncture were not significantly different in reducing chemotherapy-induced neurotoxicity and functional disability (random-effects estimates; MD: 4.30; 95% CI: −0.85~9.45; P = 0.10; I^2^ = 74%). Acupuncture was better than placebo acupuncture in reducing pain severity and pain interference with patients' daily function (fixed-effect estimates; MD: −1.14; 95% CI: 1.87 to −0.42; *P* = 0.002; I^2^ = 13%). Acupuncture was not significantly different from placebo acupuncture in relieving CIPN symptoms (MD: −0.81; 95% CI: −2.02 to 0.40, *P* = 0.19). Acupuncture improved quality of life better than placebo acupuncture (MD: 10.10; 95% CI: 12.34 to 17.86, *P* = 0.01). No severe adverse events were recorded in all five studies.

**Conclusion:**

This meta-analysis suggests that acupuncture may be more effective and safer in reducing pain severity and pain interference with patients' daily function than placebo acupuncture. Additionally, acupuncture may improve the quality of life of patients with CIPN. However, large sample size studies are needed to confirm this conclusion.

**Systematic review registration:**

https://www.crd.york.ac.uk/prospero/display_record.php?RecordID=324930, identifier: CRD42022324930.

## Introduction

Chemotherapy-induced peripheral neuropathy (CIPN) is a neurotoxic complication associated with the use of chemotherapeutic agents, such as platinum, vinca alkaloids, and taxanes (1). The incidence of CIPN is ~50% among patients treated with chemotherapeutic agents (2). Among these agents, paclitaxel, and oxaliplatin cause the highest CIPN incidence, which is as high as 80% (3). Common symptoms of CIPN include paresthesia, pain, and ataxia (4). As the dose of chemotherapeutic drugs increases, neurotoxic symptoms gradually worsen and may persist for several years with severe effects on the patient's quality of life (5, 6). The pathogenesis of CIPN has not been fully elucidated and may vary depending on the chemotherapeutic agent (7). For example, platinum agents affect neurons and mitochondrial function in the dorsal root ganglia by DNA cross-linking or oxidative stress (8, 9). Paclitaxel and vincristine, moreover, disrupt microtubule dynamics by affecting the process of depolymerization and polymerization of tubulin (10). The revised 2020 ASCO guideline does not recommend any medications for the prevention of CIPN (2). Duloxetine is the only drug recommended for treating CIPN-associated pain; however, it has limited benefits (2).

As one of the most commonly used physical therapies in Traditional Chinese Medicine, acupuncture has been used and developed for thousands of years. Currently, acupuncture is widely used in more than 160 countries. The mechanism of acupuncture is mainly related to the neural pathways and neurotransmitters/hormone factors that affect autonomic regulation, pain relief, and other therapeutics (11, 12). Several studies have been conducted on the use of acupuncture in the treatment of CIPN (13, 14). The mechanism of acupuncture in CIPN is not yet fully understood. Possible explanations are that acupuncture may relieve paclitaxel-induced neuropathic pain by mediating spinal opioid receptors, α2- and β-adrenoceptors, and promoting nerve regeneration and repair (15–18). Currently, three published systematic reviews and meta-analyses show that acupuncture may be effective and safe in the treatment of CIPN (19–21). However, due to the poor quality of the included studies, the conclusions need further substantiation. Recently, three high-quality randomized controlled trials (RCTs) on acupuncture for CIPN treatment have been reported (22–24). Two studies revealed that, compared with placebo acupuncture, acupuncture does not reduce chemotherapy-induced neurotoxicity and functional disability (22, 23). Another study showed that, compared with placebo acupuncture, acupuncture does not relieve neuropathic pain (24). The efficacy and safety of acupuncture in the treatment of CIPN remain controversial. Therefore, we aimed to compare efficacy and safety profiles between acupuncture and placebo acupuncture in the treatment of CIPN. This systematic review and meta-analysis is an updated review on this topic. Additionally, new indicators were included to assess the quality of life and CIPN symptoms.

## Methods

This systematic review and meta-analysis were conducted according to the Preferred Reporting Items for Systematic Review and Meta-Analysis (PRISMA) guidelines (25). The study's protocol was registered in PROSPERO (number CRD42022324930).

### Definitions of acupuncture

Acupuncture is defined as a form of therapy by mechanical stimulation of a specific part of the body (acupoints) (26). Based on the acupuncture treatment method, different tools, such as millineedles, fire needles, plum needles, triangular needles, and acupoint injections, are used. Among these tools, millineedles are the most widely used (27). This study focused on millineedle-based acupuncture. The millineedle-based acupuncture can be divided into manual acupuncture and electroacupuncture, based on whether manual methods or electroacupuncture instruments are used to stimulate acupuncture acupoints. The efficacy of acupuncture closely depends on the “De-Qi” feeling (soreness, numbness, heaviness, and distension). Manual acupuncture and electroacupuncture stimulate acupoints by twisting the inserted needle by hand and a stimulating current, respectively. Both manual acupuncture and electroacupuncture can make patients feel “De-Qi,” and there is no significant difference in the efficacy of the two acupuncture methods (11, 28).

### Eligibility criteria

This systematic review and meta-analysis included only RCTs. No language and publication status restrictions were instituted. Participants were diagnosed with CIPN greater than grade 1 according to the National Cancer Institute Common Terminology Criteria for Adverse Events (NCI-CTCAE 4.0) (30). Based on the intervention, participants were divided into two groups: acupuncture (treatment) and placebo acupuncture (control) groups. The placebo acupuncture procedure differed from acupuncture in that the acupuncture site was not at the acupuncture points, but closer to the acupuncture points, and the acupuncture needles touched but did not penetrate the skin, or the depth of the needles was shallow. Needle pricks and electrical stimulation were not performed. Participants did not subjectively feel “De-Qi” after acupuncture. Outcome measures included severity of neurotoxicity and associated dysfunction, severity of neuropathic pain, severity of CIPN symptoms, quality of life, and adverse events. We included studies with at least one outcome measure. The severity of neurotoxicity and associated dysfunction was evaluated on the Functional Assessment of Cancer Therapy/Gynecologic Oncology Group-Neurotoxicity (FACT/GOG-Ntx), which is used to assess sensory, motor, and hearing impairments associated with neuropathy (23). Ratings range from 0 to 44 points. Lower scores indicated more dysfunction and more severe neurotoxicity. A widely used tool for assessing neuropathic pain in patients with cancer is the Brief Pain Inventory-Short Form (BPI-SF) (31, 32). It is scored on an 11-point scale (0 = no pain; 10 = severe pain). To assess the severity of CIPN symptoms, the numerical rating scale (NRS) was used as the evaluation tool. NRS is often used to assess symptom severity in CIPN, allowing patients to assess their mean neurologic symptoms, such as tingling, numbness, and pain (0 = asymptomatic, 10 = most severe symptom imaginable) on an 11-point scale over a given day (33, 34). The European Organisation for Research and Treatment of Cancer Quality of Life Questionnaire-Core30 (EORTC QLQ-C30), which is widely used to assess the quality of life of patients undergoing cancer treatment, was the evaluation tool for quality of life (35, 36). We excluded case reports, cohort studies, reviews, conference abstracts, and no full text.

### Search strategy

Two reviewers searched PubMed, Cochrane Library, and Embase databases according to the Cochrane Handbook Guidelines (37), using the time range from inception to 1 April 2022. The search terms used were MeSH terms, such as “chemotherapy-induced peripheral neuropathy,” “acupuncture,” “placebo,” and “randomized controlled trial.” The search strategy is outlined in the supplemental search terms and strategy. Additionally, the conforming literature was found through published studies.

### Data extraction

Two researchers independently extracted the data using predesigned tabulation and checked the extraction results together. Any disagreement was resolved by a third review author. Two reviewers extracted the following information from the included literature: general characteristics of the trial (the first author's name, sample size, publication year, etc.), methods, interventions, outcomes, and adverse events. If data were unavailable, authors were contacted by phone or email for relevant information as soon as possible.

### Risk of bias assessment

Two authors independently assessed the methodological quality of the included RCTs using the risk of bias tool of the Cochrane Collaboration (38). Any disagreements were resolved by a third review author. Each study was estimated as having a high, low, or unclear risk of bias.

### Statistical analysis

All data analyses were performed using RevMan 5.3 software (The Cochrane Collaboration, Oxford, UK). Continuous data were analyzed using mean differences (MDs) with 95% confidence intervals (CIs). Dichotomous data were analyzed using risk ratio (RR) and 95% CIs. Heterogeneity was examined using the I^2^ and *Q*-test. When the heterogeneity was not statistically significant (*P* > 0.10, I^2^ <50%), the fixed-effect model was used. In cases of significant statistical heterogeneity (*P* ≤ 0.10, I^2^ >50%), a random-effects model was used. Moreover, the cause of heterogeneity was analyzed by subgroup analysis or sensitivity analysis. Subgroup analysis was performed according to different control interventions. A funnel plot was used for assessing publication bias.

### Level of evidence

The quality of the evidence was assessed for outcome measures using the Grading of Recommendations Assessment Development and Evaluation (GRADE). These five factors could reduce the quality of evidence: risk of bias, inconsistencies, inaccuracies, indirectness, and publication bias. Each of these five factors was evaluated using the GRADEpro software. The quality of the evidence was categorized into four levels: high, moderate, low, or very low quality (39, 40).

## Results

### Study selection

**Figure 1:**
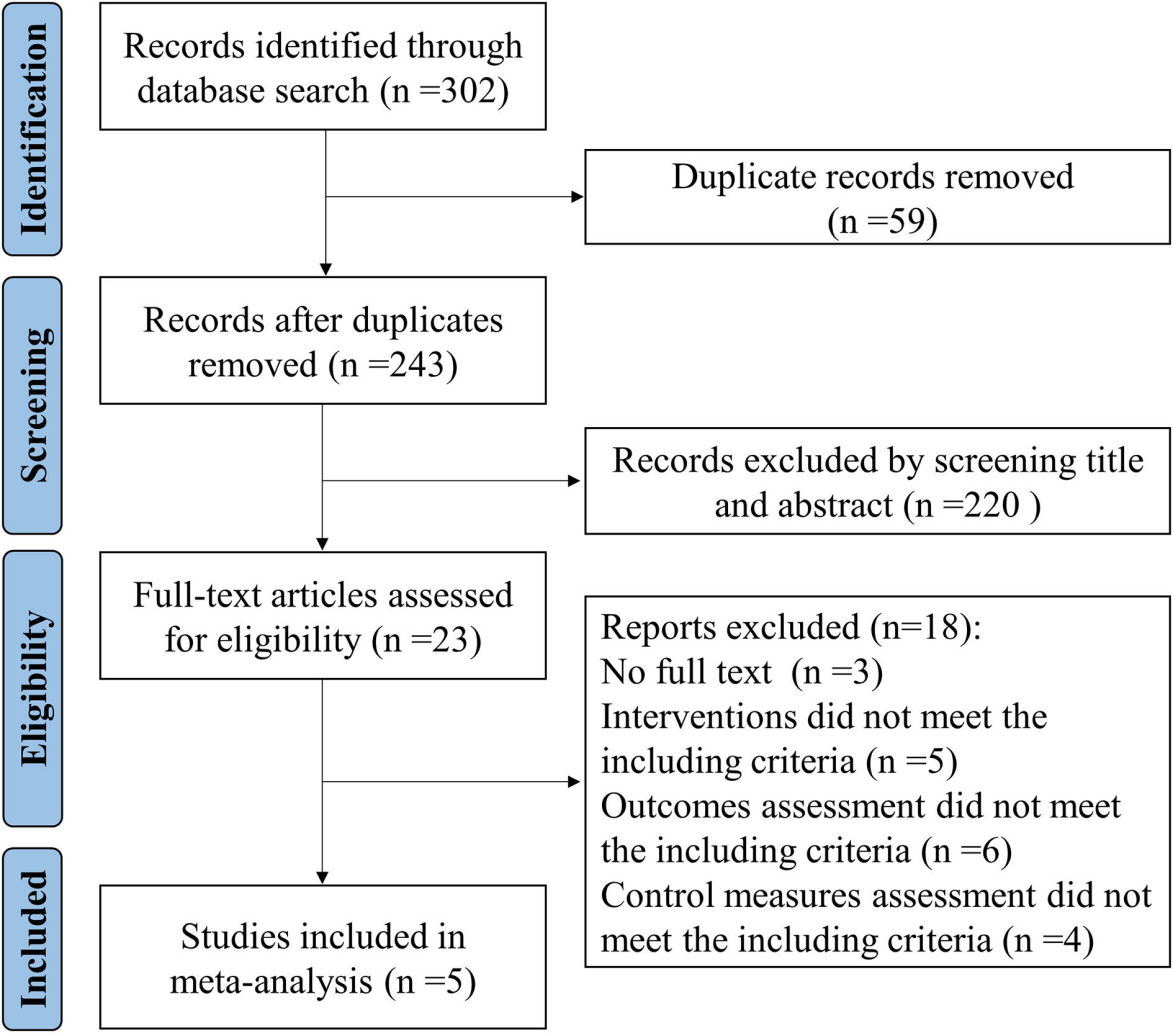
Flow chart of the study selection process.

Figure 1 shows a flowchart of the search and study selection strategies. A total of 302 records were identified by searching PubMed, Cochrane Library, and Embase databases. Fifty-nine duplicate records were identified and removed. We excluded 220 irrelevant records by screening titles and abstracts. After screening the full text, 18 trials were excluded. Finally, five studies were included.

### Study characteristics

Table 1 shows the characteristics of all five RCTs. The sample sizes ranged from 20 to 63 participants. All five studies had 225 participants altogether; and these participants had been diagnosed with CIPN. Acupuncture was used in the experimental group, while sham acupuncture was used in the control groups of four studies. One study used no treatment for the control group. For the outcome measures, three studies reported FACT-NTX and three trials reported BPI-SF. Additionally, one trial reported NRS, whereas another trial reported EORTC QLQ-C30. Four studies mentioned adverse events.

**Table 1:** The characteristics of the included trials.

**References**	**Country**	**Mean age (years)**	**Sample sizes (T/C)**	**Acupuncture**	**Control**	**Acupuncture session**	**Outcomes**	**Adverse events**
Bao et al. (23)	USA	59.70	51 (27/24)	EA	SA	Twice a week over 2 weeks and once a week over 6 weeks	FACT-NTX	T: 5
Bao et al. (29)	USA	59.70	51 (27/24)	EA	SA	Twice a week over 2 weeks and once a week over 6 weeks	NRS	Not mentioned
Greenlee et al. (13)	USA	50.00	63 (31/32)	EA	SEA	Once a week over 12 weeks	FACT-NTX; BPI-SF	T: 1
Huang et al. (22)	China	49.60	20 (10/10)	MA	SA	Twice a week over 6 weeks and once a week over 3 weeks	BPI-SF	None
Lu et al. (24)	USA	54.00	40 (20/20)	EA	NT	Three times a week over 2 weeks and twice a week over 6 weeks	FACT-NTX; BPI-SF; EORTC QLQ-C30	T: 1

USA, United States of America; EA, electroacupuncture; MA, manual acupuncture; SA, sham acupuncture; SEA, sham electroacupuncture; NT, no treatment; T, treatment group; C, control group.

### Risk of bias

**Figure 2:**
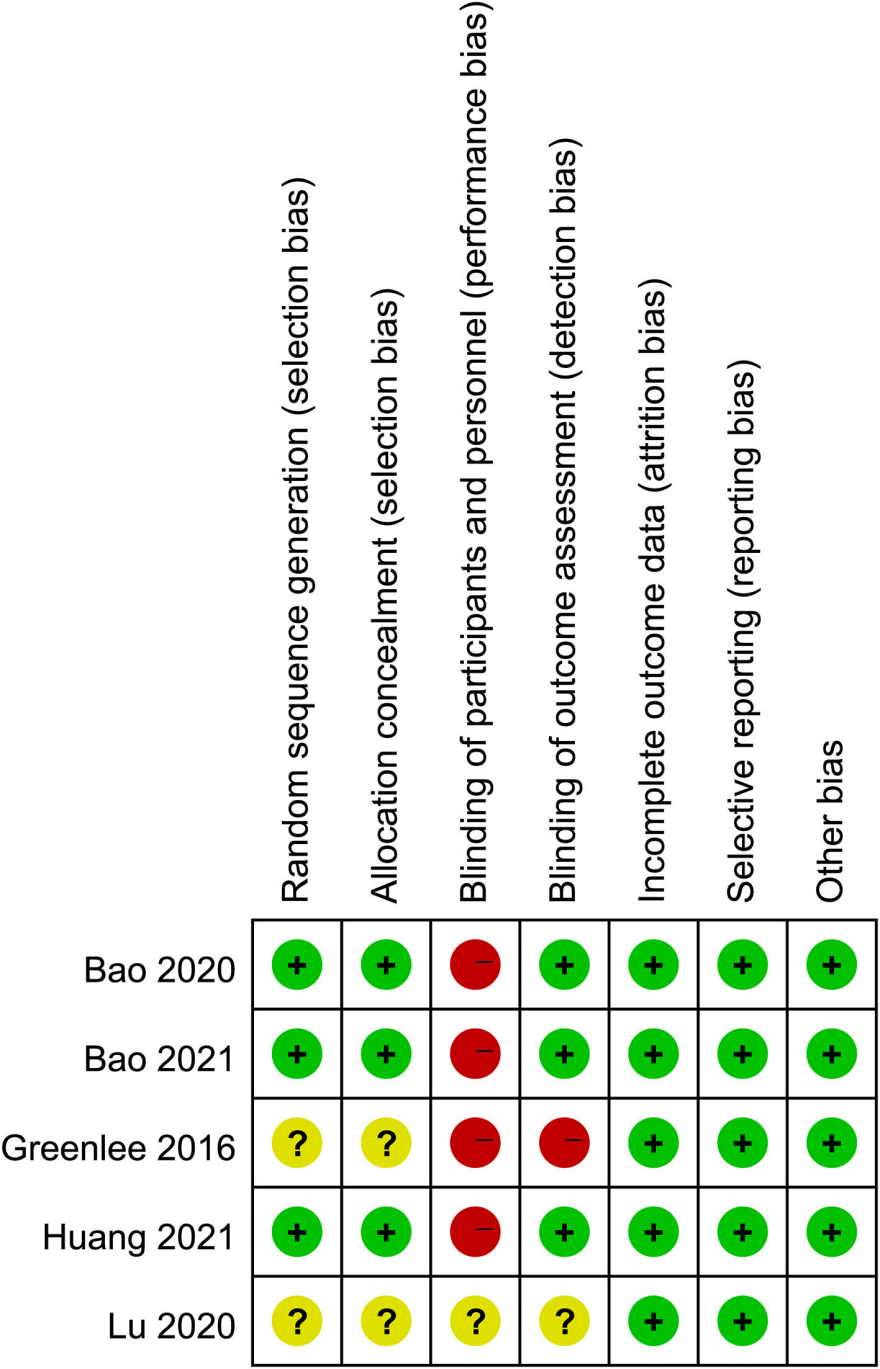
Risk of bias assessment.

**Figure 3:**
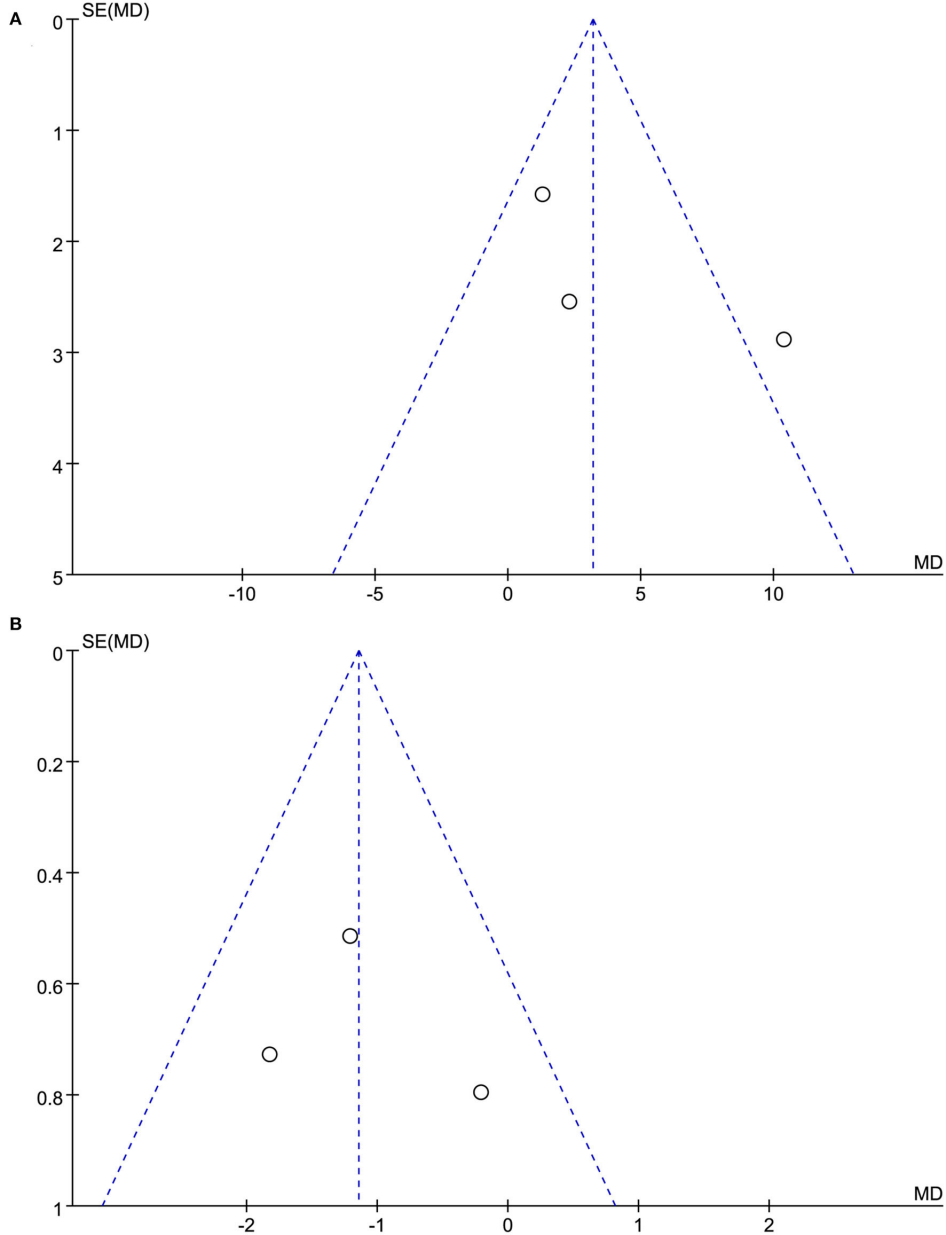
**(A)** The funnel plots of acupuncture on neurotoxicity and associated dysfunction. **(B)** The funnel plots of acupuncture on neuropathic pain.

Figure 2 shows the quality assessment of the included RCTs. Although the acupuncturists in these studies were not blinded, the overall quality of the studies was high. The funnel plots of the included studies showed no significant publication bias (Figure 3).

### Outcome measures

#### Effects of acupuncture on neurotoxicity and associated dysfunction

**Figure 4:**
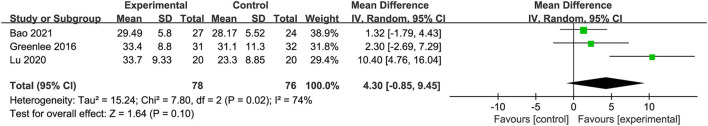
Forest plot of comparison between acupuncture and placebo acupuncture on neurotoxicity and associated dysfunction.

**Figure 5:**
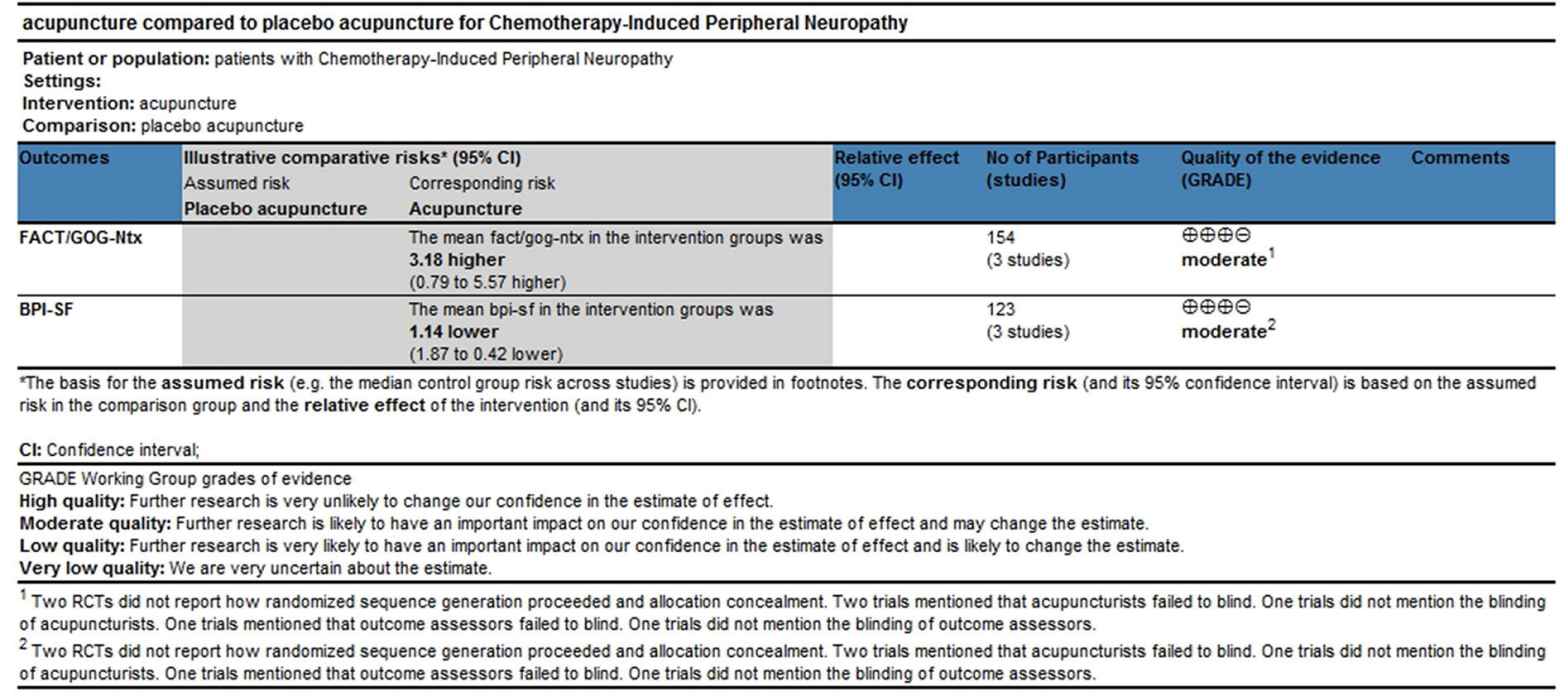
Level of evidence (GRADE).

Three trials with 154 participants reported FACT/GOG-Ntx use. A meta-analysis of three included trials revealed no statistically significant difference in the reduction of chemotherapy-induced neurotoxicity and functional disability between acupuncture and placebo acupuncture (random-effects estimates; MD: 4.30; 95% CI: −0.85 to 9.45; *P* = 0.10) with significant heterogeneity (I^2^ = 74%) (Figure 4). GRADE analysis reported that the level of evidence was moderate (Figure 5).

#### Effects of acupuncture on neuropathic pain

**Figure 6:**
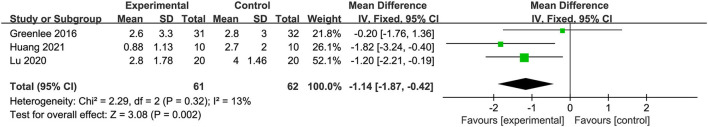
Forest plot of comparison between acupuncture and placebo acupuncture on neuropathic pain.

Three trials with 123 participants reported BPI-SF use. A meta-analysis of the three included trials showed that acupuncture was better in reducing pain severity and pain interference with patients' daily function than placebo acupuncture (fixed-effect estimates; MD: −1.14; 95% CI: 1.87 to −0.42; *P* = 0.002) with no significant heterogeneity (I^2^ = 13%) (Figure 6). GRADE analysis reported that the level of evidence was moderate (Figure 5).

#### Effects of acupuncture on CIPN symptoms

One study with 51 participants used descriptive analysis to evaluate CIPN symptoms using NRS. The results showed that acupuncture was not significantly different from placebo acupuncture in its ability to relieve CIPN symptoms (MD: −0.81; 95% CI: −2.02 to 0.40, *P* = 0.19).

#### Effects of acupuncture on quality of life

A study with 40 participants used descriptive analysis to evaluate the quality of life using EORTC QLQ-C30. The results showed that acupuncture was superior to placebo acupuncture in improving quality of life (MD: 10.10; 95% CI: 12.34–17.86, *P* = 0.01).

### Adverse events

Of the five RCTs included, four RCTs reported adverse events. A total of 174 participants were enrolled in four RCTs. Of the four RCTs, one RCT reported no adverse events, whereas three RCTs reported minor adverse events with no report of severe adverse events. One RCT reported pain at the needling site in three participants and bruising at the needling site in two participants (21). One RCT reported one case of pruritus in the feet and one point of joint pain associated with acupuncture (22). One RCT reported discomfort, minor swelling, and bruising after acupuncture needle withdrawal in one participant (11).

## Discussion

### Summary of results

Five RCTs tested the effectiveness of acupuncture in the treatment of CIPN. These placebo-controlled RCTs found that acupuncture and placebo acupuncture were not significantly different in reducing chemotherapy-induced neurotoxicity and functional disability and relieving CIPN symptoms. RCTs with placebo acupuncture as the control intervention found that acupuncture was superior to placebo acupuncture in reducing neuropathic pain severity and pain interference with patients' daily function and improving quality of life.

### Comparison with other studies and interpretation of study findings

Our findings were consistent with those of other studies in suggesting that acupuncture was better in reducing pain severity and pain interference with patients' daily function than placebo acupuncture. Additionally, we found that acupuncture may be effective in improving the quality of life among patients with CIPN. However, unlike other studies, our study found that acupuncture was not significantly different from placebo acupuncture in reducing chemotherapy-induced neurotoxicity and functional disability based on FACT/GOG-Ntx. Although both sham acupuncture and no treatment are routinely equivalent to placebo acupuncture, our study found that sham acupuncture may have a stress-induced effect on CIPN. Therefore, the results of the study may be influenced by the physiological effects of sham acupuncture on the body.

### Quality and applicability of the evidence

Although acupuncturists were not blinded, which is an essential methodological limitation, the overall quality of the studies was high. The overall level of evidence was moderate. The quality of the evidence is limited by the small sample size. However, acupuncture treatment prescriptions in these studies have a good reflection of clinical practice. In other words, acupuncture prescriptions in these studies are common prescriptions for CIPN treatments. In acupuncture treatment, most studies focus on acupuncture at the lesion site. In terms of the frequency of treatment, most studies were conducted one-to-three times a week for 9–18 sessions.

### Limitation of study

This systematic review and meta-analysis had several limitations. First, the sample sizes of the studies included in this systematic review and meta-analysis were small, as RCTs with large samples of CIPN with acupuncture are currently lacking. Second, this study did not consider the effect of different chemotherapeutic drugs, treatment doses, and tumor types on outcomes. Third, this study had significant heterogeneity in the analysis of the effects of acupuncture on neurotoxicity and associated dysfunction. Therefore, study findings should be interpreted with caution.

### Future research

In future studies, large samples and multi-center RCTs are needed to demonstrate the efficacy of acupuncture in the treatment of CIPN. Moreover, sham acupuncture may have certain biological effects, and non-invasive measures should be taken to avoid interference with the effects of sham acupuncture. Furthermore, an assessment of economic factors is necessary to inform government policy formulation.

## Conclusion

This meta-analysis suggested that acupuncture may be better and safer in reducing pain severity and pain interference with patients' daily function than placebo acupuncture. Additionally, acupuncture may improve the quality of life of patients with CIPN. However, large sample size studies are needed to confirm this conclusion.

## Data availability statement

The original contributions presented in the study are included in the article/Supplementary material, further inquiries can be directed to the corresponding author.

## Author contributions

ZX, XW, and XF contributed to the study design and manuscript revision. ZX and XW contributed to the literature search and analyzed the data. YW and CW screened the title, abstract, full texts, extracted the data, and evaluated the risk of bias. All authors read and approved the manuscript prior to submission.

## Conflict of interest

The authors declare that the research was conducted in the absence of any commercial or financial relationships that could be construed as a potential conflict of interest.

## Publisher's note

All claims expressed in this article are solely those of the authors and do not necessarily represent those of their affiliated organizations, or those of the publisher, the editors and the reviewers. Any product that may be evaluated in this article, or claim that may be made by its manufacturer, is not guaranteed or endorsed by the publisher.
