# Supplementary material for: The effectiveness and safety of acupuncture for chemotherapy-induced peripheral neuropathy: A systematic review and meta-analysis
Source: Front Neurol. 2022 Oct 3;13:963358. doi: 10.3389/fneur.2022.963358 (PMC9574072; doi:10.3389/fneur.2022.963358)
Supplement: Supplementary file 1 [file Data_Sheet_1.docx]

The Effectiveness and Safety of Acupuncture for Chemotherapy-Induced Peripheral Neuropathy: a Systematic Review and Meta-Analysis

Zhonghang Xu^1^, Xingbo Wang^1^, Yuanyu Wu^1^, Chengbing Wang^1^, Xuedong Fang^1^

^1^China-Japan Union Hospital of Jilin University, Jilin University, Changchun, China

Correspondence**:** Xuedong Fang

Department of Gastrointestinal Colorectal and Anal Surgery, China-Japan Union Hospital of Jilin University, No.126 Xiantai Street, Changchun, China.

Email: [fangxd@jlu.edu.cn](mailto:fangxd@jlu.edu.cn).

**Supplementary Material**

**1.Supplementary search terms and strategies**

**Table1. Search strategy used in PubMed database.**

| Number | Search items |  |
| --- | --- | --- |
| 1  2  3  4  5  6  7  8  9  10  11  12  13  14  15  16  17  18  19 | Chemotherapy-Induced Peripheral Neuropathy.MeSH  CIPN.ti,ab  Chemotherapy Induced Neurotoxicity.ti,ab  Chemically-Induced Disorders.ti,ab  Peripheral Neuropathy.ti,ab  or 1-5  Acupuncture.MeSH  Manual Acupuncture.ti,ab  Electroacupuncture.ti,ab  Body Acupuncture.ti,ab  Acupuncture Therapy.ti,ab  or 7-11  Randomized Controlled Trial.pt  Controlled Clinical Trial.pt  Randomized.ti,ab  Trials.ti,ab  Placebo.ti,ab  or 13-17  6 and 12 and 18 | |

**Table2. Search strategy used in Cochrane Library**

| Number | Search items |  |
| --- | --- | --- |
| 1  2  3  4  5  6  7  8  9  10  11  12  13 | Chemotherapy-Induced Peripheral Neuropathy.MeSH  CIPN.ti,ab  Chemotherapy Induced Neurotoxicity.ti,ab  Chemically-Induced Disorders.ti,ab  Peripheral Neuropathy.ti,ab  or 1-5  Acupuncture.MeSH  Manual Acupuncture.ti,ab  Electroacupuncture.ti,ab  Body Acupuncture.ti,ab  Acupuncture Therapy.ti,ab  or 7-11  6 and 12 | |

**Table3. Search strategy used in Embase**

| Number | Search items |  |
| --- | --- | --- |
| 1  2  3  4  5  6  7  8  9  10  11  12  13  14  15  16  17  18  19 | Chemotherapy-Induced Peripheral Neuropathy.MeSH  CIPN.ti,ab  Chemotherapy Induced Neurotoxicity.ti,ab  Chemically-Induced Disorders.ti,ab  Peripheral Neuropathy.ti,ab  or 1-5  Acupuncture.MeSH  Manual Acupuncture.ti,ab  Electroacupuncture.ti,ab  Body Acupuncture.ti,ab  Acupuncture Therapy.ti,ab  or 7-11  Randomized Controlled Trial.pt  Controlled Clinical Trial.pt  Randomized.ti,ab  Trials.ti,ab  Placebo.ti,ab  or 13-17  6 and 12 and 18 | |
